# Peer Acceptance Protects Global Self-esteem from Negative Effects of Low Closeness to Parents During Adolescence and Early Adulthood

**DOI:** 10.1007/s10964-013-9929-1

**Published:** 2013-02-24

**Authors:** Marianne Skogbrott Birkeland, Kyrre Breivik, Bente Wold

**Affiliations:** 1 Department of Health Promotion and Development, The Faculty of Psychology, University of Bergen, Postboks 7807, 5020 Bergen, Norway; 2 Regional Centre for Child and Youth Mental Health and Child Welfare, Uni Health, Uni Research, Postboks 7810, 5020 Bergen, Norway

**Keywords:** Longitudinal, Measurement invariance, Parent relationships, Peer relationships, Self-esteem, Self-worth

## Abstract

Having a distant relationship with parents seems to increase the risk of developing a more negative global self-esteem. This article describes a longitudinal study of 1,090 Norwegian adolescents from the age of 13–23 (54 % males) that explored whether peer acceptance can act as a moderator and protect global self-esteem against the negative effects of experiencing low closeness in relationships with parents. A quadratic latent growth curve for global self-esteem with closeness to parents and peer acceptance as time-varying covariates was modeled, taking partial measurement invariance in global self-esteem into account. Peer acceptance was found to have a general protective effect on global self-esteem for all adolescents. In addition, at most ages, peer acceptance was found to have a protective-stabilizing effect on the relationship between closeness to parents and global self-esteem. This indicates that peer acceptance can be an especially valuable source of global self-esteem when closeness to parents is low.

## Introduction

Global self-esteem, defined as the “positive or negative attitude toward a particular object, namely, the self” (Rosenberg 1965, p. 30), has been found to become somewhat more positive during adolescence before stabilizing in young adulthood (Huang 2010; Trzesniewski et al. 2003). The individual trajectories of global self-esteem may be predicted by adolescents’ feelings of closeness to and acceptance from significant others, such as parents and peers (Leary et al. 1998). Even though adolescents often spend less time with their parents than they did when they were children, close and supportive relationships with their parents are still important sources of positive self-esteem (Mattanah et al. 2011). However, some adolescents do not have close relationships with their parents during adolescence and young adulthood. In these cases, being accepted by peers may be especially beneficial in the development of global self-esteem. This study investigates whether peer acceptance actually can protect against the negative effects of experiencing low closeness to parents on global self-esteem during adolescence and young adulthood (13–23 years).

### Closeness to Parents and Global Self-esteem

Attachment theory (Bowlby 1969) emphasized the strong emotional bond between parents and children and proposed that the relationship between a child and her/his significant others provides the basis for the child’s working model of herself/himself. A child who experiences parents who are emotionally available, loving and supportive of the child’s attempts to master the world may construct a working model of herself/himself as valuable and competent. Having close relationships with parents also has been found to be associated with positive global self-esteem in adolescence (Laursen and Collins 2009; Mattanah et al. 2011).

The emotional context of parenting has been found to be crucial for positive development among children and adolescents (Steinberg 2001). During adolescence, this emotional bond may be manifested in mutual respect and understanding, shared activities, and self-disclosure. However, disagreements between parents and adolescents are common, as adolescents may experience less companionship and intimacy with parents (Buhrmester and Furman 1987), and the level of negative affect in parent–child conflict may be higher during adolescence than during other age periods (Laursen and Collins 2009). Furthermore, poorly managed conflicts have been found to be associated with more negative global self-esteem (Caughlin and Malis 2004). Most parents are able to adjust to the adolescents’ changing needs, and the conflicts between parents and adolescents do typically not represent a threat to relationships, but some families with a history of interpersonal problems may lack the adaptive patterns needed for developing new forms of closeness (Laursen and Collins 2009). In these cases, positive relationships with others, such as peers, may reduce the negative impact that low-quality relationships with parents may have on the adolescents’ global self-esteem (Steinberg 2001).

### Peer Acceptance as a Protective Factor

During adolescence, peer relationships become more salient. Adolescents spend increasingly more time with peers, often without supervision from adults, and expectations of opinions of peers come to have a more important value to them (Brown and Larson 2009). Being accepted in friendship groups and reputation-based crowds are important to solidify adolescents’ social and personal identity (Brown et al. 1994). Being accepted in specific social groups with high status is highly valued (Eder 1985) and is sometimes pursued at the cost of intimate friendships with unpopular peers. Perceived popularity may have direct effects on global self-esteem, which is not mediated by the supportiveness of friendships (Litwack et al. 2012). One of the major functions of peers in adolescence may be to support the individuation processes related to developing independence from parents and developing a separate identity, which increases the relevance of belonging to a peer group and being accepted by peers (Rubin et al. 2006). Consequently, being accepted by peers may be crucial for maintaining a positive global self-esteem during adolescence.

One of the main ideas in the research on resilience is that protective factors may buffer against the negative effects of adversity. In Luthar et al.’s terminology (Luthar et al. 2000), both the main effect and interaction effects are viewed as protective factors. If peer acceptance has a main effect on global self-esteem, peer acceptance serves as a generally protective effect that is ameliorative for all adolescents, regardless of their closeness to parents.

In addition to having a general protective effect, peer acceptance may also moderate effects of other processes on global self-esteem. A moderator is a variable that affects the direction and/or strength of the relationship between a predictor variable and an outcome variable, resulting in an interaction effect between the predictor and moderator variables (Baron and Kenny 1986). The present article examines peer acceptance as a possible moderator of the association between closeness to parents and global self-esteem.

General protective effects (main effects) can be distinguished from interactive or moderating protective effect by using more specific terms that describe different types of moderating protective effects, such as “protective-stabilizing,” “protective but reactive,” and “protective-enhancing” (Luthar et al. 2000). A protective-stabilizing effect occurs when a protective factor contributes to stabilize individuals’ positive functioning in the face of risk. Thus, peer acceptance can be said to have a protective-stabilizing effect if peer acceptance fosters similar levels of global self-esteem across levels of closeness to parents. Thus, peer acceptance can act as a buffer and stabilize children’s global self-esteem in the face of a lack of closeness to parents. For example, low levels of closeness to parents may be associated with loneliness, depressive symptoms and fewer opportunities to learn social skills, which may be associated with a negative development of global self-esteem. Experiencing acceptance in a peer group may buffer against this effect by offering an arena where the adolescent belongs, can make friends and use social skills not acquired at home, which may increase positive global self-esteem. Furthermore, when parents are distant or not emotionally available, peers can provide positive feedback and concrete help. This may maintain the adolescents’ feelings about their positive value as persons, and keep their working models of attachment stable.

We are not aware of any studies that have explicitly examined whether peer acceptance moderates the relationship between closeness to parents and global self-esteem, which mean that we had to draw upon studies of similar phenomena. Some studies have found protective-stabilizing effects of different aspects of relationships with peers on global self-esteem or internalizing problems. For example, in a study of maltreated adolescents, friendship quality was found to have a protective-stabilizing effect on global self-esteem development (Bolger et al. 1998). Another study found that peer acceptance acted as a buffer against developing internalizing problems when the adolescents were rejected by parents (Sentse et al. 2010).These studies indicate that peer acceptance may have a protective-stabilizing effect on global self-esteem when closeness to parents is low.

Another possibility may be that peer acceptance fosters positive global self-esteem across levels of risk, but is particularly effective under low levels of risk. This implies that peer acceptance may be generally protective, but less so when children/adolescents have little closeness with their parents (protective but reactive interaction effect). Perhaps being accepted by peers is generally positive for global self-esteem, but adolescents may not benefit fully from being accepted by peers if they do not have close relationships with their parents. This may be because low levels of positive social relationship experiences with their parents also may put adolescents in a defensive position in other social relationships, for example, with peers, which can limit what the adolescent is able to gain from being accepted by peers. We are not aware of any studies that directly support a protective but reactive effect of peer acceptance on global self-esteem in the face of low closeness to parents. However, a study that examined the potential moderating effect of peer support on the negative impact of having a low adult support (from parents, teachers, and neighbors) on psychological well-being found that being supported by peers had a weaker protective effect as the degree of adult support decreased (Buchanan and Bowen 2008).

Finally, peer acceptance may facilitate higher global self-esteem in the face of adversity and thus may in fact serve to increase global self-esteem as risk increases (protective-enhancing interaction effect). Thus, high peer acceptance may allow adolescents with low closeness to their parents to learn and develop, with global self-esteem increasing as adolescents’ relationships with their parents deteriorate. In this case, high peer acceptance makes it possible to benefit from lower closeness to parents by providing a safe arena in which to process difficult experiences with parents and to learn from these experiences in a way that entails higher global self-esteem. Perhaps distant relationships with parents can encourage at least some adolescents to take more responsibility for themselves and feel better about themselves, as long as they have the second arena where are accepted.

There is also some support for the view that peer acceptance may have such a protective-enhancing effect. One study found that whereas little use of positive reinforcement from parents (“positive parenting”) was associated with internalizing problems among early adolescents with low quality friendships, the same potential risk factor led to slightly lower internalizing problems among their peers who reported high levels of friendship quality (Gaertner et al. 2010).

The evidence of possible moderating effects of aspects of peer relationships on the association between aspects of relationships with parents and psychological well-being is mixed. One explanation of these different findings may be that the studies use different types of samples who experience very different levels of closeness to parents. For example, Bolger et al. (1998) used a sample of maltreated adolescents. In addition, the protective effects could depend on age and gender. Most of the earlier studies used samples of young adolescents, and there is a lack of studies comparing these relationships across time in a community sample of adolescents.

### Differences in the Protective Effect of Peer Acceptance Across Age

Different relationships may have different functions at different points in development. For example, a study found that whereas parents were seen as the most frequent providers of support during early adolescence, friends and romantic partners were perceived as more supportive than parents during later adolescence (Furman and Buhrmester 1992). Friendships appear to play an increasingly important role with increasing age during adolescence (Rubin et al. 2006), which may indicate that being accepted by peers may have a stronger general protective effect among older adolescents than younger adolescents. In addition, while family relationships are the most important relationships during childhood, gradually other relationships, with friends and romantic partners, come to serve many of the same functions (Collins et al. 2007). If peers can serve some of the same functions as parents during later adolescence, global self-esteem may not be as strongly dependent on closeness to parents as in early adolescence. Thus, peer acceptance may both have a stronger effect on global self-esteem in general, and also may have a stronger moderating effect on the relationship between closeness to parents and global self-esteem among older than younger adolescents.

### Gender Differences

Most studies have found gender differences in levels of global self-esteem (Kling et al. 1999), but the relationship between closeness to parents and global self-esteem may still be similar across gender. Parental support has been found to predict global self-esteem similarly across both genders (Rueger et al. 2010), and a meta-analysis revealed similar relationships between parental attachment and global self-esteem during college for both genders (Mattanah et al. 2011). Moreover, the studies of neither Sentse et al. (2010) nor Gaertner et al. (2010) found gender differences in the interaction effects between relationships to peers and aspects of relationships with parents, on internalizing problems. Therefore, the potential protective effect of peer acceptance on the relationships between having a less close relationship with parents and negative self-esteem are expected to be similar for males and females.

## The Present Study

The literature to date suggests that closeness to parents and peer acceptance are important for adolescents’ global self-esteem. Some studies also suggest that, to some degree, peer acceptance may buffer the negative effect on global self-esteem of experiencing lower closeness with parents. No previous study has, however, focused on differences in a possible protective effect of peer acceptance across the course of adolescence. The present study contributes to the field by conducting a 10-year prospective study of a community sample of Norwegian adolescents and examines associations between closeness to parents and peer acceptance, and global self-esteem, when controlling for the development of global self-esteem.

The main research question was: To what extent do peer acceptance and closeness to parents have direct ameliorative effects on global self-esteem, and how can any possible moderating effects of peer acceptance on the association between closeness to parents and global self-esteem across adolescence and young adulthood best be described? Based on the previous literature, closeness to parents and peer acceptance were expected to have protective direct effects on global self-esteem for all adolescents and young adults. It, furthermore, was expected that the protective function of peer acceptance would increase somewhat with age, particularly among adolescents with low closeness to their parents (protective-stabilizing effect). The associations between closeness to parents, peer acceptance and global self-esteem were hypothesized to be similar across gender.

## Method

### Sample and Design

A representative sample of seventh graders (mean age 13.3 ± 0.3 years) and their parents in the county of Hordaland in western Norway was invited to participate in the Norwegian Longitudinal Health Behaviour Study during the fall of 1990. This study was originally conducted to examine social influences on adolescents’ health behaviours, but a wide range of questions about psychological well-being was also included. The baseline sample consisted of students from 22 secondary schools randomly selected from the total population of secondary schools in the county (100 schools), and the parents of 927 adolescents (78 % of the total sample) provided written informed consent. No data on race or ethnicity exist, but because Hordaland county in Norway was a rather homogeneous society, it was assumed that almost all participants were of Norwegian and Caucasian origin. This sample was then followed up eight times through their adolescence and young adulthood. The present study used the data from 1990 (age 13), 1992 (age 15), 1995 (age 18) and 2000 (age 23). The data were collected through self-administered questionnaires delivered at school in 1990 and 1992, and by mail in 1995 and 2000. During the first data collections at school, any new students in the classes were invited to participate, which increased the total number of pupils who participated at least once to 1,242. To increase the possibility for reaching as many as possible of the participants, two reminders were sent out to participants who did not respond. Strict procedures were followed to ensure confidentiality and the Norwegian Data Inspectorate approved the study.

### Measures

#### Global Self-Esteem

Global self-esteem was measured with a revised version of Rosenberg’s Self-esteem Scale called the Global Negative Self-Evaluation Scale (Alsaker and Olweus 1986). This scale is adapted for use with adolescents (Alsaker and Olweus 1986). The six items were: “At times I think I am no good at all” (item 1), “I would like to change many things about myself” (item 2), “All in all, I am inclined to feel that I am a failure” (item 3), “I feel I do not have much to be proud of” (item 4), “I have often wanted to be someone else” (item 5), and “I certainly feel useless at times” (item 6). The response categories ranged from “applies exactly” (1) to “does not apply at all” (6). Cronbach’s alphas at ages 13, 15, 18, and 23 were 0.86, 0.90, 0.88, and 0.92, respectively. This scale is comparable to well-known scales of global self-esteem, such as Rosenberg’s and Harter’s scales (Alsaker and Olweus 1986). In the present study, the Global Negative Self-Evaluation Scale was assumed to measure the underlying concept of global self-esteem, which was modeled as a latent variable. To ensure that the scale was operating in the same way across gender and time, testing for measurement invariance was completed (see below).

#### Closeness to Parents

Closeness to parents was measured by the items: “My mother and I understand each other well,” “My father and I understand each other well,” “My parents praise and encourage me,” “There is good cohesiveness in my family,” and “I enjoy myself when I am with my parents.” The response categories for three of the items ranged from “applies exactly” (6) to “does not apply at all” (1), and for the remaining two ranged from “very often” (6) to “seldom or never” (1). These items were averaged and centered. Cronbach’s alphas at the ages of 13, 15, 18 and 23 years were 0.83, 0.84, 0.85, and 0.86, respectively.

#### Peer Acceptance

Peer acceptance was measured by two items, one concerning peers in general: “I am doing fine with others of my age,” and “My peers seem to like me.” The six response categories ranged from “applies exactly” (6) to “does not apply at all” (1). These items were averaged and centered. The correlations between the items at the ages of 13, 15, 18 and 23 years were 0.67, 0.76, 0.82, and 0.82, respectively.

#### Living Arrangements

At every time point, the participants were asked: “Who do you live with?” and provided with a list of possible living arrangements.

### Statistical Analyses

All data analysis and modeling were performed with Mplus Version 6.1 (Muthén and Muthén 2007). To correct for the somewhat skewed distributions in global self-esteem, maximum likelihood estimation with robust errors (MLR) was applied. To determine model fit, Chi squared (χ^2^), degrees of freedom, MLR correction factor, root mean square error of approximation (RMSEA), and comparative fit index (CFI) were assessed. Values of RMSEA <0.05 and values of CFI above 0.95 were considered to denote a well-fitting model (Browne and Cudeck 1992; Hu and Bentler 1999).

As recommended by Dimitrov (2010), before doing growth curve analyses, measurement invariance analyses were conducted to determine whether the instrument measuring global self-esteem operated in the same way across gender and time. In the baseline configural model, items were constrained to load on the same factor across time. By fixing the factor means to zero, model parameterization and factor scaling were achieved. Furthermore, the residuals of the similarly worded items measured at different time points were allowed to correlate. In addition, preliminary confirmatory factor analyses revealed a local dependency of item 2 and item 5, which was probable given the similar wording of the items. Thus, the residuals of items 2 and 5 were allowed to correlate with one another at every time point.

To test for weak invariance across gender and time, the factor loadings were first constrained to be equal across gender, followed by time. Similarly, when testing for strong invariance, item intercepts were constrained to be equal across gender, followed by time. Differences between nested models were evaluated by assessing both differences in CFI and Satorra–Bentler scaled χ^2^ difference tests (adjusted for MLR). ∆CFI above 0.002 was considered to be non-ignorable (Meade et al. 2008).

**Fig. 1:**
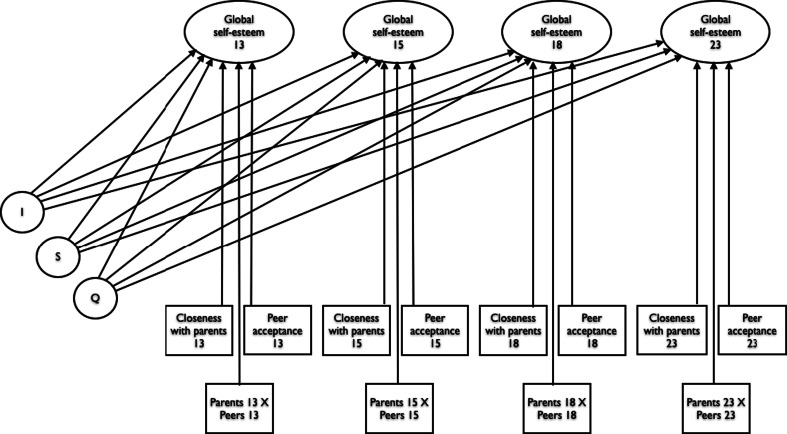
The conditional quadratic trajectory model with time-varying covariates

After partial measurement invariance was established, a multigroup unconditional growth curve model was modeled and fitted, with groups based on gender. Then, the time-varying covariates were added to the growth curve model. Figure 1 provides a graphical illustration of the growth curve model with time-varying covariates.

### Missing Data Analysis

Of the 1,242 participants in the study, 152 did not respond on any of the relevant measures. The total sample consisted of 591 males and 499 females, making the total N = 1,090. 134 of the males and 171 of the females provided complete data on all items across the four time points. The percent of missing data across all variables ranged from 0 % (gender) to 55 % (“My peers seem to like me” at age 23). Not surprisingly, most of the missing data seem to be explained by higher wave nonresponse (participants who dropped out of the study) at the later waves. A one-way between subjects ANOVA was conducted to compare levels of global self-esteem, closeness to parents and peer acceptance at age 13 among participants who dropped out of the study before 2000 from participants who did not drop out. There were no significant differences in neither global self-esteem [F(1,839) = 1.93, *p* = 0.16], closeness to parents [F(1,779] = 3.48, *p* = 0.06], nor peer acceptance [F(1,804) = 0.71, *p* = 0.40].

Full information maximum likelihood (FIML) estimation with robust standard errors was used to handle missing data. This approach assumes data are missing at random (MAR), and all observed information is used to produce the maximum likelihood estimation of parameters. This is one of the best approaches currently available to handle missing data (Acock 2005).

## Results

### Description of Sample and Measurement Invariance Models of Global Self-esteem

Table 1 shows that most adolescents lived with both their parents at age 13 and 15. At age 23, most young adults had left home.

**Table 1:** Description of sample: age, number and living arrangements

Age	13	15	18	23
N	924	963	963	627
Living with both parents (%)	83	82	71	15
Living with only mother (%)	11	12	13	
Living with only father (%)	2	2	3	
Not living with parents (%)	4	3	14	85

The model fit statistics for various degrees of measurement invariance are shown in Table 2. The configural invariance measurement model fitted the data well. When measurement variance was constricted across gender and time, the fit decreased somewhat. The final model fit for the partial strong invariance across gender and time was χ^2^(499, N = 1,089) = 806.534, *p* < 0.05, MLR correction factor = 1.168, CFI = 0.965, RMSEA = 0.034, Δχ = 38.466 (1), *p* > 0.05, which is in line with the recommendations by Byrne et al. (1989) was regarded as acceptable.

**Table 2:** Competing model fits for tests of measurement invariance across gender and time

Model	χ	df	Corr	RMSEA	CFI	Model	ΔCFI	Δchi
1. Configural model	699.908	448	1.163	0.032	0.971			
2. Weak invariance across gender	714.370	468	1.171	0.031	0.972	M2-M1	0.001	16.690 (20)
3. Weak invariance across gender and time	799.309	483	1.175	0.035	0.964	M3-M2	0.008	78.982 (15)**
4. Partial weak invariance across gender and time^a^	725.455	479	1.173	0.031	0.972	M4-M2	0.000	11.471 (11)
5. Strong invariance across gender	757.982	499	1.166	0.031	0.970	M5-M4	0.002	32.903 (20)*
6. Partial strong invariance across gender^b^	752.311	498	1.167	0.031	0.971	M6-M4	0.001	26.570 (19)
7. Strong invariance across gender and time	1,079.069	516	1.162	0.045	0.936	M7-M6	0.035	367.240 (18)**
8. Partial strong invariance across gender and time^c^	806.534	499	1.168	0.034	0.965	M8-M6	0.006	38.466 (1)**

* *p* < 0.05, ** *p* < 0.01

^a^Invariance was relaxed at item 4 at age 23, item 5 at age 13, and item 6 at age 15 and 23

^b^Invariance was relaxed across gender at item 2 at age 13

^c^Invariance was relaxed at item 2 at age 15 and 18, item 3 at age 13 and 23, item 4 at age 13 and 15, item 5 at age 13 and 15, and item 6 at age 15 and 18. In addition,at age 13, invariance at item 2, 3, 4, 5 and 6 was relaxed across gender, and at age 15, invariance at item 2 and 4 was relaxed across gender, and at age 23, item 3 invariance was relaxed across gender

The unstandardized factor loadings for the final model generally did not show any systematic patterns with regard to magnitude across gender and age, which means that the items loaded similarly on the latent variable when measured at different ages. However, item 5 has a somewhat high loading at age 13, and item 6 has a somewhat low loading at age 23 in comparison with other time points. These findings indicate that wanting to be someone else is more reflective of low global self-esteem at age 13, whereas feeling useless is less reflective of low global self-esteem at age 23.

Furthermore, inspection of intercept estimates indicated that the unstandardized intercepts increased over time for many of the items, especially from age 18 to age 23. This may indicate that the adolescents with the same level of latent factor global self-esteem tended to respond with higher scores when they were 23 than when they were 13 years old. One exception to this pattern was found—the intercepts of item 6 (“I certainly feel useless at times”) decreased somewhat at ages 15 and 18 before increasing at age 23. Thus, these analyses indicate that some of the included items functioned somewhat differently over time, which was taken into account in the growth curve analyses.

### Unconditional Latent Growth Model of Global Self-esteem

To establish the pattern of change in global self-esteem from ages 13 to 23, a linear unconditional growth curve for each gender was estimated, taking the partial measurement invariance into account. The resulting model provided a good fit to the data: χ^2^(506, N = 1,089) = 824.796, *p* < 0.05, MLR correction factor = 1.168, CFI = 0.964, RMSEA = 0.034. Adding a quadratic term added significantly to the model fit: χ^2^(504, N = 1,089) = 797.318, *p* < 0.05, MLR correction factor = 1.168, CFI = 0.967, RMSEA = 0.033, ΔCFI = 0.003, Δχ = 27.478 (2), *p* < 0.05. The model was trimmed by fixing the nonsignificant variance (random estimates) of quadratic slope to zero for both genders and fixing the nonsignificant quadratic slope among males to zero. This resulted in a good model fit: χ^2^(504, N = 1,089) = 798.144, *p* < 0.05, MLR correction factor = 1.168, CFI = 0.967, RMSEA = 0.033.

**Fig. 2:**
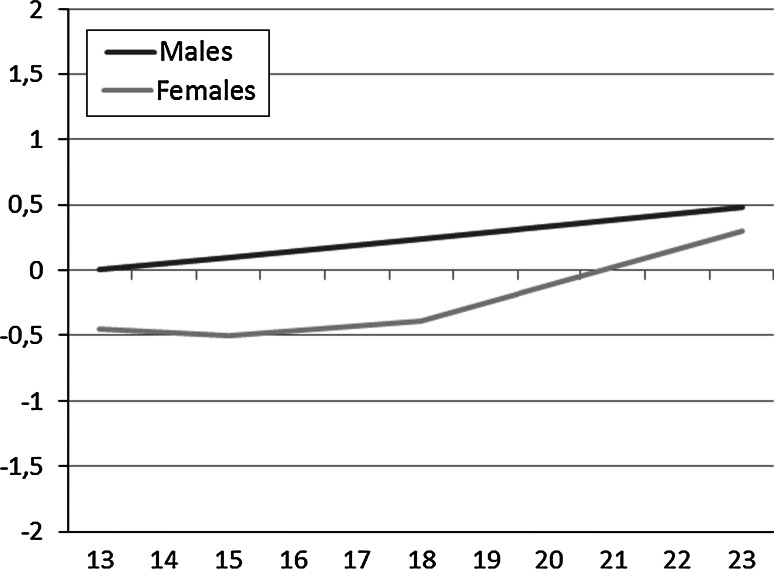
The unconditional growth curve for development of global self-esteem among males and females from age 13 to age 23. Standard deviation is used as unit of measurement, and males’ global self-esteem at age 13 is constrained to 0

The parameter estimates of the fixed and random effects can be seen in Table 3. The estimates for males showed a linear slope, reflecting an increasing trajectory of global self-esteem. Among females, the parameter estimates indicate a significantly lower baseline of global self-esteem at age 13 than the males’ baseline. Furthermore, the parameter estimates for females indicate a nonsignificant negative linear slope, and a significant positive quadratic slope, which reflects a U-shaped trajectory. Mean trajectories of males’ and females’ global self-esteem can be seen in Fig. 2. Significant random effects in both intercepts and slopes indicate significant individual variability in both the starting point (intercept) and rate of change (slopes).

**Table 3:** Unconditional growth curve models of global self-esteem development from age 13 to age 23

Effect	Males		Females	
	Est	SE	Est	SE
Fixed				
Intercept	0.000	0.000	−0.448**	0.079
Slope	0.498**	0.076	−0.505	0.269
Quadratic slope	0.000	0.000	1.246**	0.243
Random				
Intercept	0.686**	0.082	0.704**	0.095
Slope	0.833**	0.215	0.815**	0.254
Quadratic slope	0.000	0.000	0.000	0.000

* p < 0.05, ** *p* < 0.01

### Latent Growth Model with Time-Varying Covariates

Controlling for the latent growth curve, global self-esteem at each time point was regressed on the centered covariates of closeness to parents and peer acceptance, and their interaction terms. This model provided a good fit to the data: χ^2^(1,059, N = 1,089) = 1,536.168, *p* < 0.05, MLR correction factor = 1.110, CFI = 0.953, RMSEA = 0.029. Further, to test whether the model could be more parsimonious, the effects of the time-specific measures were constrained across gender and time. The competing model fits can be seen in Table 4. Constraining across gender did not affect model fit (Model 2), but constraining across time resulted in a significantly poorer model fit (Model 3). Inspection of the modification indices indicated that the interaction between closeness to parents and peer acceptance among females at age 13 was responsible for Model 3’s poorer model fit. Modification of the model by specifying a separate estimate for this interaction term (Model 4) was successful in achieving a model fit that did not differ significantly from Model 2. Thus, Table 4 shows that with the exception of the interaction between closeness to parents and peer acceptance among females at age 13, all estimates can be constrained across both gender and time without the model fit deteriorating.

**Table 4:** Competing model fits of parsimonious models for effects from time-specific covariates on time-specific global self-esteem beyond growth trajectory

Model	χ	df	Corr	RMSEA	CFI	Model	ΔCFI	Δchi
M1. Par + peer + interactions	1,536.168	1,057	1.100	0.029	0.953			
M2. Constrained across gender	1,555.297	1,069	1.102	0.029	0.952	M2-M1	−0.001	18.896 (12)
M3. Constrained across time	1,581.029	1,078	1.104	0.029	0.950	M3-M2	−0.002	23.494 (9)**
M4. PaXPe13 relaxed for females	1,567.014	1,077	1.104	0.029	0.952	M4-M2	−0.000	11.702 (8)

**Fig. 3:**
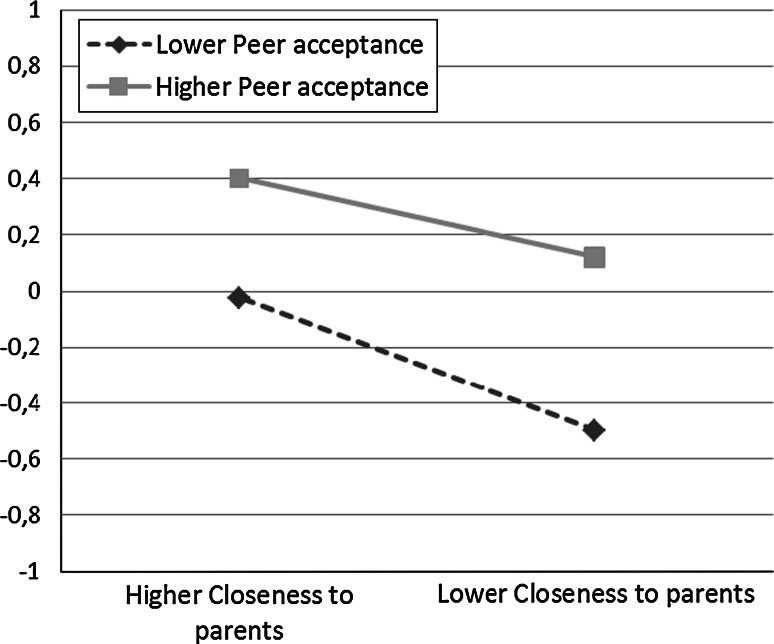
Interaction *plot* for associations between closeness to parents and peer acceptance on global self-esteem for males at age 13. Interaction* plots* for both genders aged 15–23 show similar patterns. Higher/lower peer acceptance and closeness to parents represent one standard deviation over and under mean

**Fig. 4:**
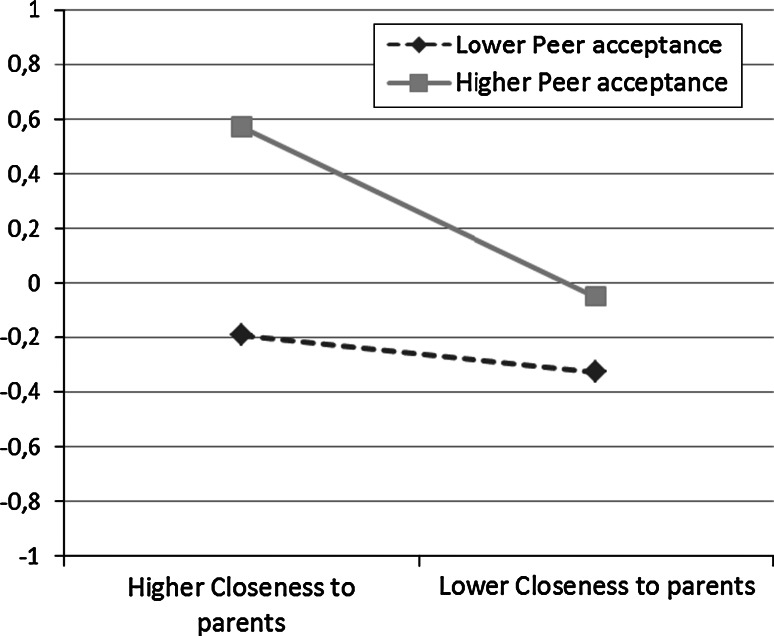
Interaction* plot* for associations between closeness to parents and peer acceptance on global self-esteem for females at age 13. Higher/lower peer acceptance and closeness to parents represent one standard deviation over and under mean

The final unstandardized estimates of the associations of the time-specific covariates with time-specific global self-esteem beyond the growth trajectory can be seen in Table 5. The results show that closeness to parents has a positive and stable significant association (0.21) with global self-esteem, and that peer acceptance has a positive and stable significant association (0.30) with global self-esteem, for both genders at all time points. At most time points, a significant negative interaction (−0.06) of the associations between closeness to parents and peer acceptance with global self-esteem among both genders was found. Figure 3 shows an example from males at age 13, and indicates that among adolescence with higher peer acceptance, global self-esteem is more stable across closeness to parents. The one exception to this pattern was among females at age 13, where the estimated interaction term was 0.15 between closeness to parents and peer acceptance on global self-esteem. Figure 4 shows this interaction, and indicates that among adolescence with higher peer acceptance, closeness to parents is particularly strongly associated with global self-esteem.

**Table 5:** Unstandardized estimates for effects of time-specific covariates on time-specific global self-esteem beyond growth trajectory, parsimonious model (M4)

Unstandardized	Global self-esteem 13		Global self-esteem 15		Global self-esteem 18		Global self-esteem 23	
	Est	SE	Est	SE	Est	SE	Est	SE
Males and females								
Closeness to parents	0.213**	0.022	0.213**	0.022	0.213**	0.022	0.213**	0.022
Peer acceptance	0.296**	0.025	0.296**	0.025	0.296**	0.025	0.296**	0.025
Parent × peer	Males: −0.063**	0.023	−0.063**	0.023	−0.063**	0.023	−0.063**	0.023
	Females: 0.147**	0.060						

* *p* < 0.05, ** *p* < 0.01

## Discussion

Global self-esteem is strongly connected to the quality of social relationships (Leary et al. 1998). These relationships change character during adolescence as individuals may experience less intimacy with parents (Buhrmester and Furman 1987), and higher levels of negative affect in parent–child conflicts (Laursen and Collins 2009). Peers may become more important social partners than before (Brown and Larson 2009), and being accepted by peers may reduce the negative impact that low-quality relationships with parents may have on the adolescents’ global self-esteem (Steinberg 2001). This was the rationale for examining relationships between closeness to parents, peer acceptance and global self-esteem across adolescence and young adulthood (13–23 years).

The results obtained in the present study confirmed that low closeness to parents increases the risk for more negative global self-esteem. Furthermore, the results supported the hypothesis that peer acceptance has a protective effect on global self-esteem for all adolescents and young adults. However, the hypothesis that this protective effect would increase with age was not supported. In addition, among most adolescents and young adults, peer acceptance may have a small protective-stabilizing effect, meaning that high peer acceptance may stabilize global self-esteem when closeness to parents is low. Thus, the present study contributes to the literature by demonstrating that both closeness to parents and peer acceptance are associated with global self-esteem during adolescence and young adulthood (13–23 years), and that peer acceptance may buffer the negative effects of low closeness to parents on global self-esteem.

### Associations Between Social Relationships and Global Self-Esteem

The course of global self-esteem across adolescence was consistent with the findings of earlier studies that have indicated that global self-esteem is quite stable, but improves slightly during adolescence (Birkeland et al. 2012; Erol and Orth 2011; Huang 2010). Generally, males reported higher mean levels of positive global self-esteem than females, and their global self-esteem improves steadily from 13 to 23 years. Among females, global self-esteem was quite stable until the age of 18, and then improved substantially from 18 to 23 years. One explanation why females seem to have more negative global self-esteem around age 18 may be that body image issues, which have been found to be strongly related to global self-esteem (Van den Berg et al. 2010), may be more prevalent in females. Females may experience higher body shame and body surveillance than males (Knauss et al. 2008), and this may be especially evident around the age 18, where many females establish romantic relationships and have their sexual debut. Still, most adolescents of both genders seem to have relatively positive feeling about themselves across adolescence and young adulthood.

For the great majority of the adolescents, the importance of closeness to parents and peer acceptance for global self-esteem was stable from age 13 to age 23. The importance of closeness to parents for global self-esteem did not decrease during adolescence, which may indicate that being close to parents is still important, even though this closeness might be manifested differently. Most young adults have left home before age 23, but the association between closeness to parents and global self-esteem seems not to be influenced by this. This confirms earlier findings from earlier research that indicate that parents are still important sources of global self-esteem also in adolescence and young adulthood (Laursen and Collins 2009; Mattanah et al. 2011).

Contrary to the hypothesis that peer acceptance may have an increasing protective effect on the potential negative impact of having a less close relationship with parents on global self-esteem, the protective effects of peer acceptance did not increase during adolescence or young adulthood. This may reflect that peer acceptance is important for all adolescents regardless of age. That feeling accepted by peers is equally important across time may indicate that this is a psychological need that do not change even though social relationships change character during adolescence.

The results showed that both being accepted by peers and closeness to parents were positively related to global self-esteem for all adolescents. Among adolescents of both genders from age 15 to age 23, and among 13-year-old males, the detrimental effect of having a nonoptimal relationship with their parents was lower among adolescents with high peer acceptance, which suggests that being accepted by peers may buffer against the negative effects of distant relationships with parents on global self-esteem. This protective-stabilizing effect is in line with findings from some other studies (Bolger et al. 1998; Sentse et al. 2010). However, in the present study, the buffering effect was relatively small, perhaps because the sample consisted of a community sample of adolescents with relatively high levels of closeness to parents rather than special samples like a sample of maltreated adolescents. In addition, the protective-stabilizing effect could have been somewhat stronger if other aspects of peer relationships than simply being accepted had been used. It is not unreasonable to expect that intimacy with peers, for example, might have had a stronger stabilizing effect.

Among the 13-year-old females, a protective but reactive effect was found; the protective effect of peer acceptance seemed to decrease as the closeness to parents decreased. For these females, having a certain level of closeness to parents may be a prerequisite to acquiring the full benefits of peer acceptance. The reason why this appeared only among the youngest females may be grounded in gender role socialization. Whereas adolescent females may be socialized to seek emotional closeness in their relationships with their parents, adolescent males may be encouraged to be more independent at an earlier age (Leaper et al. 1998; Operario et al. 2006).

### Strengths and Limitations

The present study has several strong strengths. Among them is the 10-year longitudinal data set, which was analyzed using sophisticated methods. Partial measurement invariance in global self-esteem was taken into account, and the growth curve was controlled for before assessing the associations between closeness to parents and peer acceptance and global self-esteem. In contrast to other studies, the present study used a community sample and included aspects of relationships with both parent and peers in the same study. This made it possible to test whether the time-specific measures of global self-esteem were related to time-specific measures of closeness to parents and peer acceptance beyond the influence of the trajectory process underlying global self-esteem. Thus, the study was well suited to explore the relationships between closeness to parents, peer acceptance and global self-esteem.

One limitation of the present study is that all measurement instruments were self-reported and may suffer from the effect of social desirability. In addition, the measure of global self-esteem is not able to separate healthy global self-esteem from narcissistic global self-esteem. Furthermore, a considerable amount of the data was missing because of dropouts. Finally, despite the longitudinal design, it is not possible to draw conclusions regarding the direction of effects and causality between social relationships and global self-esteem.

### Implications and Conclusion

In this study, it was found that peer acceptance seems to moderate the potential negative impact of having a less close relationship with parents on global self-esteem. One of the implications of these findings from this study is that interventions aimed at increasing the well-being of adolescents and young adults can benefit from including both parents and peers. Parents can be educated about the importance of, and means of achieving, close relationships with their children throughout adolescence and into young adulthood. School and organized leisure-time activities may be suitable arenas for interventions directed toward identifying and building on supportive and accepting relationships with peers that may protect against the negative effects of difficulties in other relationships.

This study adds to the literature by demonstrating that closeness to parents and peer acceptance were stably and positively associated with global self-esteem from 13 to 23 years. Another pivotal finding of the present study was that peer acceptance can have a protective-stabilizing effect on global self-esteem when relationships with parents are less close. Future studies need to further explore how this can be translated into interventions.
